# Characterization of uncommon portosystemic collateral circulations in patients with hepatic cirrhosis

**DOI:** 10.3892/ol.2014.2626

**Published:** 2014-10-22

**Authors:** QIN WU, LIJUN SHEN, JINDONG CHU, XUEMEI MA, BO JIN, FANPING MENG, JINPIN CHEN, YANLING WANG, LIBING WU, JUN HAN, WENHUI ZHANG, WEI MA, HUAMING WANG, HANWEI LI

**Affiliations:** Liver Cirrhosis Therapeutic Center, 302 Hospital of the People’s Liberation Army, Beijing 100039, P.R. China

**Keywords:** liver cirrhosis, collateral circulation, hepatic encephalopathy, portal hypertension, esophageal varicosity, gastric fundus varicosity

## Abstract

The purpose of the present study was to characterize uncommon portosystemic collateral circulation in hepatic cirrhosis. Portosystemic uncommon collateral circulation (UCC) was detected, characterized and evaluated by a combination of spiral computed tomography angiography, three-dimensional imaging angiography and electronic gastroscopy in patients diagnosed with hepatic cirrhosis. In total, 118 cases with UCC were detected from a pool of 700 hepatic cirrhosis patients with portal hypertension. The incidence was 16.86% and included cases with splenic-renal, gastro-renal, paravertebral, retroperitoneal, gastric-splenic and cardio-phrenic angle vein shunts. The occurrence rate of UCC formation increased with the Child-Pugh grade. Compared with common collateral circulations, the incidence of severe esophageal or gastric fundus varicose veins, severe portal hypertensive gastropathy and the incidence of a large quantity of ascites was much lower in the patients with UCC (P<0.01), whereas the incidence of hepatic encephalopathy and chronic elevated blood ammonia levels was significantly higher (P<0.01). The incidence of uncommon portosystemic collateral circulation is extremely common in patients with liver cirrhosis and is associated with the Child-Pugh grades of hepatic function. UCC can aid in the relief of the complications derived from portal hypertension, but it may increase the incidence of hepatic encephalopathy and chronic elevated blood ammonia levels.

## Introduction

Portosystemic collateral circulation is a consequence of portal hypertension, which occurs in chronic liver disease and is responsible for numerous complications, including hemorrhage resulting from the rupture of esophageal and fundal gastric varices and hepatic encephalopathy (1–3). Due to cirrhosis of the liver, the blood flow in the portal veins becomes blocked or stenosis leads to blood stasis, which induces markedly higher pressure within the portal veins, and subsequently results in an extrahepatic portosystemic shunt (4). Once portal hypertension develops, it dramatically alters the extrahepatic vascular beds in the splanchnic and systemic circulation, leading to arterial vasodilation and collateral circulation formation, which diverts a greater proportion of the blood flow into the portal vein. Portal hypertension is exacerbated by the increased portal blood flow, which can also be replicated in canine models (5,6).

The hemodynamic alterations of blood flow in cirrhosis include portal hypertension and hyperdynamic circulation. Portal hypertension development comprises three pathophysiological components, namely, intrahepatic, systemic (splanchnic) and collateral circulation (7,8). In general, portosystemic collateral circulation can be classified into uncommon collateral circulation (UCC) and common collateral circulation (CCC). CCC has been studied for a long time, and includes esophageal and gastric varices, abdominal and umbilical vein dilation and hemorrhoidal vein dilation (8–11). However, there are few notable studies on UCC, including splenorenal, gastric renal, retroperitoneal and cardiac angle venous shunts. In the present study, the features and clinical findings of UCC in patients with hepatic cirrhosis were characterized using multi-slice computed tomography (CT) scanning, three-dimensional (3D) reconstruction, electronic gastric endoscopy and liver function evaluations.

## Materials and methods

### Subjects

Between January 2010 and June 2012, 700 patients who suffered from liver cirrhosis and were treated in the 302 Hospital of the People’s Liberation Army (Beijing, China) were recruited for the present study. The patients consisted of 496 males and 204 females, aged between 15 and 78 years old [mean (±standard deviation), 49.7±10.6], all with complete medical records. All the patients underwent endoscopy and abdominal 64-slice spiral CT, their medical histories were taken, and physical and imaging examinations were performed, which allowed for a clinical diagnosis of liver cirrhosis using criteria described by Dooley *et al* (12). The enrolled patients consisted of 449 cases of liver cirrhosis due to hepatitis B virus (HBV) infection, 63 cases of liver cirrhosis due to hepatitis C virus (HCV) infection, 53 cases of alcoholic cirrhosis, 41 cases of undetermined etiology, 30 cases of primary biliary cirrhosis, 24 cases of autoimmune liver cirrhosis, 13 cases of Budd-Chiari syndrome, nine cases of liver cirrhosis due to HBV/HCV coinfection, five cases of liver degeneration, four cases of drug-induced liver cirrhosis, four cases of overlap syndrome, two cases of hepatic veno-occlusive disease, two cases of portal vein spongiform degeneration and one case of cardiac cirrhosis. The liver function of the patients was classified in accordance with the Child-Pugh classification standard (5), identifying 227 cases as grade A, 302 cases as grade B and 171 cases as grade C. Written informed consent was obtained from all patients and was approved by the ethics committee of 302 Hospital of the People’s Liberation Army (Beijing, China).

### Methods

The patients underwent a physical examination conducted by specialist physicians, and blood, liver and kidney function, blood ammonia, autoantibody series, serum copper, ceruloplasmin, hepatitis biomarker and coagulation tests were also performed. Electronic gastroscopy was performed by experts at the 302 Hospital of the People’s Liberation Army on an Olympus Q260 type gastroscope (Olympus, Tokyo, Japan). Abdominal helical CT scans were conducted using a 64-slice CT scanner (GE Healthcare, Cleveland, OH, USA) and the images were analyzed using AW4.5 working platform Volume Viewer 5-D post-processing software for 3D vascular reconstruction (GE Healthcare). The diagnosis was made by two experienced radiologists.

### Grouping

The patients were divided into either the CCC or UCC group. The CCC group consisted of patients with the presence of esophageal varices or attached umbilical vein dilation only, excluding the simultaneous formation of UCC. Patients were assigned into the UCC group as long as there was evidence of the formation of collateral circulation, including the simultaneous presence of gastroesophageal varices and simple UCC.

### Statistical analysis

All data are expressed as the mean ± standard deviation, and were analyzed using the Chinese High Intellectualized Statistical Software 2010 (Yuanyitang Science & Technology, Beijing, China). Qualitative data were examined using the χ^2^ test, whereas quantitative data (one-way ordinal data) were examined using the Wilcoxon test or Kruskal-Wallis test. P<0.05 was considered to indicate a statistically significant difference.

## Results

### Incidence of esophageal and gastric varices

Examination by gastroscopy revealed that the incidence of esophageal and gastric varices was 78.00% (546/700) overall, and 67.40% (153/227), 85.10% (257/302) and 79.53% (136/171) at Child-Pugh grades A, B and C, respectively (Table I).

### Incidence of UCC

Following examination of the abdominal CT scans and 3D reconstruction imaging, 118 of the total 700 cases (16.86%) were diagnosed with UCC. As shown in Table II, the most frequent types of UCC were splenorenal and gastric-renal vein shunts (Table II).

### Association between UCC and Child-Pugh classification

The development of UCC was revealed to be closely associated with the Child-Pugh classification of liver function. As shown in Table III, the incidence rate of UCC for Child-Pugh grades A, B and C was 10.13% (23/227), 19.21% (58/302) and 21.64% (37/171), respectively.

### Association between UCC and esophageal or gastric varices

The incidence of esophageal and gastric varices was 83.90% (99/118) in the UCC group and 87.24% (383/439) in the CCC group, with no significant difference between the two groups. However, in the UCC group, the incidence of moderate/severe esophageal and gastric varices was significantly lower compared with the CCC group (Table IV).

### Association between UCC and portal hypertensive gastropathy

The incidence of portal hypertensive gastropathy in the UCC group was lower (59.32%; 70/118) compared with the CCC group (67.43%; 296/439), yet there was no statistically significant difference between the two groups. However, among the UCC cases, the rate of moderate/severe portal hypertensive gastropathy was significantly lower than in the CCC patients (Table V).

### Association between UCC and ascites

As shown in Table VI, the incidence of ascites in the UCC group was 44.07% (52/118), which was lower than that of the CCC group (53.99%; 237/439), but the difference was not statistically significant. However, the incidence of a moderate or large amount of ascites in the UCC group was significantly lower compared with the CCC group (Table VI).

### Association between UCC/CCC and high blood ammonia levels

Certain UCC cases possessed an elevated blood ammonia level, and the incidence (35.59%; 42/118) was much higher compared with the CCC group (18.45%; 81/439) (Table VII).

## Discussion

The present results revealed that in the 700 cases of liver cirrhosis, the incidence of esophageal and gastric varices was 78.00%, which is higher than has previously been reported. The incidence of patients with esophageal or gastric varices with a Child-Pugh classification of A, B or C was 67.40, 85.10 and 79.53%, respectively. These results are consistent with studies previously reported in the literature (7,9,13,14).

Following the development of portal hypertension and liver cirrhosis, blood from the portal vein system is diverted from the liver, then countercurrent flow occurs and the circulation of the body is inundated via the collateral vessels, thus multiple portosystemic pathways develop (1,3,13). This process results in the following most commonly observed varicose veins from various portosystemic pathways: Esophageal and gastric varices induced by the gastric left and short gastric veins; varicose veins of the abdominal wall induced by the umbilical vein and the affiliated abdominal umbilical veins; and the formation of a venous plexus of the rectum induced by the inferior mesenteric vein (7,9). The worse the classification of the liver function following cirrhosis, the higher the rate of esophageal and gastric varices (14). With the development of hepatic cirrhosis, if the aforementioned multiple portosystemic pathways fail to divert blood flow to relieve the rise in the portal pressure, a number of UCCs develop to further relieve the pressure in the portal vein.

The present study indicated that the incidence of UCC was 16.86%. In addition, the incidence of UCC increased with the grade of Child-Pugh classification (A–C). The incidence of UCC at grade A, B and C was 10.13, 19.21 and 21.05%, respectively. This suggests that the formation of UCC is closely associated with the extent of hepatic cirrhosis, as the higher the degree of hepatic cirrhosis, the higher the portal venous pressure and the incidence of UCC.

Splenorenal shunt (50.00%) and stomach renal vein shunts (42.37%) were the two most common types of UCC in the present study. The occurrence of UCC may relieve the traffic of blood flow, thus reducing portal pressure and greatly mitigating the complications induced by portal hypertension. For example, the incidences of moderate/severe esophageal and gastric varices and portal hypertensive gastropathy among the patients with UCC were significantly lower than in the patients with CCC, which partly compromised the risk of bleeding in these patients. More significantly, following the formation of UCC in the patients, moderate or massive ascites were less developed, to a certain extent, thereby reducing the risk of infection and ascites-induced pain in the patients. However, the rate of hepatic encephalopathy reached 35.59% among the patients with UCC, which was significantly higher compared with the patients with CCC.

It is conjectured that, due to the formation of UCC, the portal blood flow is increased and a large proportion of ammonia and other metabolites are directly diverted into the systemic circulation. By contrast, the blood flow that perfuses the liver is decreased, thereby leading to an exacerbation of hepatic ischemia and impaired liver function. The impairment of liver function also diminishes the ability to detoxify ammonia and other metabolites, thereby increasing the risk of hepatic encephalopathy induced by portosystemic shunt (15,16).

In clinical practice, the treatment of portal hypertension remains a formidable challenge due to sophisticated pathogenic conditions, risk factors and surgical difficulties. Scaling the balance of the aforementioned advantages and disadvantages of UCC provides extremely important guidance for the treatment of portal hypertension. It is recommended that these patients do not undergo the surgical procedure of portosystemic shunt, as this establishes an abnormal connection between the portal vein and the systemic circulation, allowing portal blood to reach the systemic circulation without passing through the liver first, which causes the liver to be deprived of hepatotrophic factors, which may result in hepatic atrophy and encephalopathy. Performing endoscopic sclerotherapy, without prior knowledge of whether the patient possesses gastric shunts, may easily lead to sudden patient mortality due to ectopic embolization. In terms of surgical or interventional treatment, without a comprehensive understanding of the UCC of the patient, the procedure may inadvertently damage blood vessels and cause severe bleeding. Therefore, attention should be paid to the presence of UCC in liver cirrhosis patients.

In conclusion, uncommon portosystemic collateral circulation commonly occurs in patients with liver cirrhosis, and is associated with the Child-Pugh grade of hepatic function. UCC can aid in relieving the complications derived from portal hypertension, but may increase the incidence of hepatic encephalopathy and chronic elevated blood ammonia levels. The present study recommends clinical vigilance for doctors whilst treating the complications of portal hypertension.

## Figures and Tables

**Table I:** Association between esophageal and/or gastric varices and C-P functional classification (cases).

	Classification of liver function			
		
Group	C-P A	C-P B	C-P C	Total
With CCC	153	257	136	546
Without CCC	74	45	35	154
Total	227	302	171	700

P<0.01. CCC, common collateral circulation; C-P, Child-Pugh.

**Table II:** Classification and proportion of UCC.

UCC, shunt	Incidence, n (%)	Ratio (%)
Splenorenal	59 (8.43)	50.00
Gastric-renal	50 (7.14)	42.37
Paravertebral	3 (0.43)	2.54
Retroperitoneal	2 (0.29)	1.69
Gastric-splen	2 (0.29)	1.69
Cardiac angle vein	2 (0.29)	1.69
Total	118 (16.86)	100.00

UCC, uncommon collateral circulation.

**Table III:** Association between uncommon collateral circulation and C-P classification (cases).

	Classification of liver function			
		
Group	C-P A	C-P B	C-P C	Total
UCC	23	58	37	118
CCC	133	195	111	439
None	71	49	23	143
Total	227	302	171	700

Kruskal-Wallis Method: Hc=26.320; P=0.000. C-P, Child-Pugh; CCC, common collateral circulation; UCC, uncommon collateral circulation.

**Table IV:** Association between UCC and esophageal and/or gastric varices (cases).

	Esophagus and/or gastric varices				
		
Group	None	Slight	Moderate	Severe	Total
UCC	19	34	35	30	118
CCC	56	85	135	163	439
None	79	24	23	17	143
Total	154	143	193	210	700

Kruskal-Wallis method: Hc=92.722; P=0.000. CCC, common collateral circulation; UCC, uncommon collateral circulation.

**Table V:** Association between UCC and portal hypertensive gastropathy (cases).

	Portal hypertensive gastrophy				
		
Group	None	Slight	Moderate	Severe	Total
UCC	48	38	25	7	118
CCC	143	122	111	63	439
None	60	48	24	11	143
Total	251	208	160	81	700

Kruskal-Wallis method: Hc=13.247; P=0.000. CCC, common collateral circulation; UCC, uncommon collateral circulation.

**Table VI:** Association between uncommon collateral circulation and ascites (cases).

	Ascites				
		
Group	None	A little	Moderate	Large	Total
UCC	66	30	11	11	118
CCC	202	93	69	75	439
None	82	35	14	12	143
Total	350	158	94	98	700

Kruskal-Wallis method: Hc=13.273; P=0.001. CCC, common collateral circulation; UCC, uncommon collateral circulation.

**Table VII:** Association between UCC/CCC and hepatic encephalopathy or chronic elevated blood ammonia level (cases).

	Elevated blood ammonia level or hepatic encephalopathy		
		
Group	Without	With	Total
UCC	76	42	118
CCC	358	81	439
None	130	13	143
Total	564	136	700

χ^2^=29.7271; P=0.000. CCC, common collateral circulation; UCC, uncommon collateral circulation.
